# Comparative Efficacy of Rivaroxaban and Immunoglobulin Therapy in the Treatment of Livedoid Vasculopathy: A Systematic Review

**DOI:** 10.7759/cureus.28485

**Published:** 2022-08-27

**Authors:** Shivana Ramphall, Swarnima Rijal, Vishakh Prakash, Heba Ekladios, Jiya Mulayamkuzhiyil Saju, Naishal Mandal, Nang I Kham, Rabia Shahid, Shaili S Naik, Sathish Venugopal

**Affiliations:** 1 Internal Medicine, California Institute of Behavioral Neurosciences & Psychology, Fairfield, USA; 2 Psychiatry, California Institute of Behavioral Neurosciences & Psychology, Fairfield, USA; 3 Internal medicine, California Institute of Behavioral Neurosciences & Psychology, Fairfield, USA

**Keywords:** complement-mediated, fibrin thrombi, immunoglobulin therapy, rivaroxaban, chronic coagulation disorder, atrophie blanche, livedoid vasculopathy

## Abstract

Livedoid vasculopathy (LV) is an uncommon chronic coagulation disorder whose underlying etiology is not yet fully understood. It predominantly affects females, especially those in late adolescence. There is currently limited research on treatment options for those with this diagnosis. The present systematic review aims to compare the efficacy of rivaroxaban and intravenous immunoglobulin (IVIG) therapy in the treatment of livedoid vasculopathy. A detailed search was conducted from April 20, 2022, to May 1, 2022, using four databases: Elsevier, Medline Complete, Medline Ovid, and PubMed. Out of these, 20 relevant articles were used, and the data was extracted and analyzed. Both rivaroxaban and IVIG were shown to be effective treatment options with similar treatment response times. However, future large-scale clinical trials are needed to determine an established treatment regimen for these patients.

## Introduction and background

Livedoid vasculopathy (LV) is a rare thrombotic skin disorder that challenges the diagnostic and therapeutic skills of the medical community. It is well established that LV is the consequence of a coagulation disorder, and it is distinct from inflammatory vasculitis [1]. LV was once thought to be vasculitis, but increasing consensus shows that alterations in the local or systemic coagulation control mechanism cause fibrin thrombi to form in the superficial cutaneous vessels [2]. The condition has been documented in patients who have factor V Leiden mutations, protein C deficiency, antiphospholipid antibody syndrome, elevated plasma homocysteine levels, abnormalities in fibrinolysis, and enhanced platelet activation, despite the fact that the underlying etiology is unknown [3].

LV first appears as painful and/or itchy erythematous, purpuric plaques, or papules on one or both sides of the ankles. Atrophie blanche, or atrophic stellate white scars, may emerge when these lesions are inflamed for a period of three to four months [4]. Low tissue perfusion frequently results in poor wound healing and inefficient microbe eradication by leukocytes; hence, increasing the risk of infection [5]. The incidence of livedoid vasculopathy is believed to be one in 100,000 people, with a clear female predilection (female/male ratio of 3:1). The average onset age is 45 years. The condition usually manifests itself in late adolescence, up to the age of 30 [6].

When LV is suspected, a thorough history, dermatological examination, and laboratory work-up are required to exclude other conditions in the differential diagnosis [2]. A skin biopsy specimen for histopathological examination is required to diagnose LV. In the dermis, the histology of LV is characterized by dilated and tortuous blood vessels. The vascular wall is thicker and oedematous due to endothelial cell growth. Some vessels exhibit fibrin deposition inside both the vessel wall and the lumen [7]. Typical histopathological findings include hyalinized degeneration of the subintimal layer of superficial cutaneous arteries accompanied by intraluminal fibrin deposits, intraluminal thrombosis, red blood cell extravasation, and little perivascular lymphocytic infiltration [2]. Immunofluorescence may demonstrate immunoglobulin (IgG and IgM) and complement (C3) in the vessel walls [8].

LV offers a significant therapeutic challenge to the treating physician because of the absence of multicenter trials and the disease's low prevalence. Thus far, therapy has always been an individual treatment effort with off-label usage [6]. Aspirin, dipyridamole, subcutaneous heparin, and pentoxifylline are the most often utilized first therapies [9]. Anticoagulants such as warfarin, subcutaneous heparin, and tissue plasminogen activator have since been employed with moderate effectiveness by researchers. These drugs are difficult to give or need regular monitoring, which often leads to a reduction in patient compliance [10]. Recent studies have shown that rivaroxaban, which needs less monitoring, or intravenous immunoglobulin (IVIG) treatment, which has few side effects, is effective. 

This is a systematic review of clinical trials, observational studies, retrospective studies, case series, and case reports to compare two treatment modalities and their efficacy in improving vasculopathy symptoms. The goal is to paint a clearer picture of which of these two drugs, rivaroxaban and IVIG, can clinically improve patient outcomes and to add to the limited research that is currently present.

## Review

Methods

A systematic literature search was undertaken in accordance with the Preferred Reporting Items for Systematic Reviews and Meta-Analyses (PRISMA) criteria. Free and paid full-text publications indexed in PubMed, Elsevier, Medline Complete, and Medline Ovid were searched from April 20, 2022, to May 1, 2022, using the keywords "Livedoid vasculopathy," "Immunoglobulins," "Rivaroxaban," and "Livedoid vasculopathy therapy." Table 1 provides the comprehensive search technique using four data sources.

**Table 1:** Search strategy using keywords in Elsevier, Medline Complete, Medline Ovid, and PubMed

S.No	Databases	Keywords	Search results
1.	Elsevier	Livedoid vasculopathy AND Immunoglobulins	179
2.	Elsevier	Livedoid vasculopathy AND Rivaroxaban	69
3.	Medline Complete	Livedoid vasculopathy AND Immunoglobulins	23
4.	Medline Complete	Livedoid vasculopathy AND Rivaroxaban	16
5.	Medline Ovid	Livedoid vasculopathy treatment	5190
6.	PubMed	Livedoid vasculopathy treatment	149

After the search was completed, duplicates were found and removed by two reviewers. The relevant publications were identified by examining the titles and abstracts. Articles published in English up to December 2021 were included. Biopsy-proven LV, combination treatment with other medications (as long as only one drug is present), all etiologies of LV, and the word "atrophie blanche" were all included. Case reports, case series, randomized controlled trials (RCTs), correspondence studies, observational studies, and retrospective studies were also included. The age range of the patient population was 17-80 years. Other types of vasculitis with ulcers, other types of ulcers associated with other diseases, and the term "livedoid vasculitis" were excluded. Animal studies were excluded from the review.

Results

**Figure 1:**
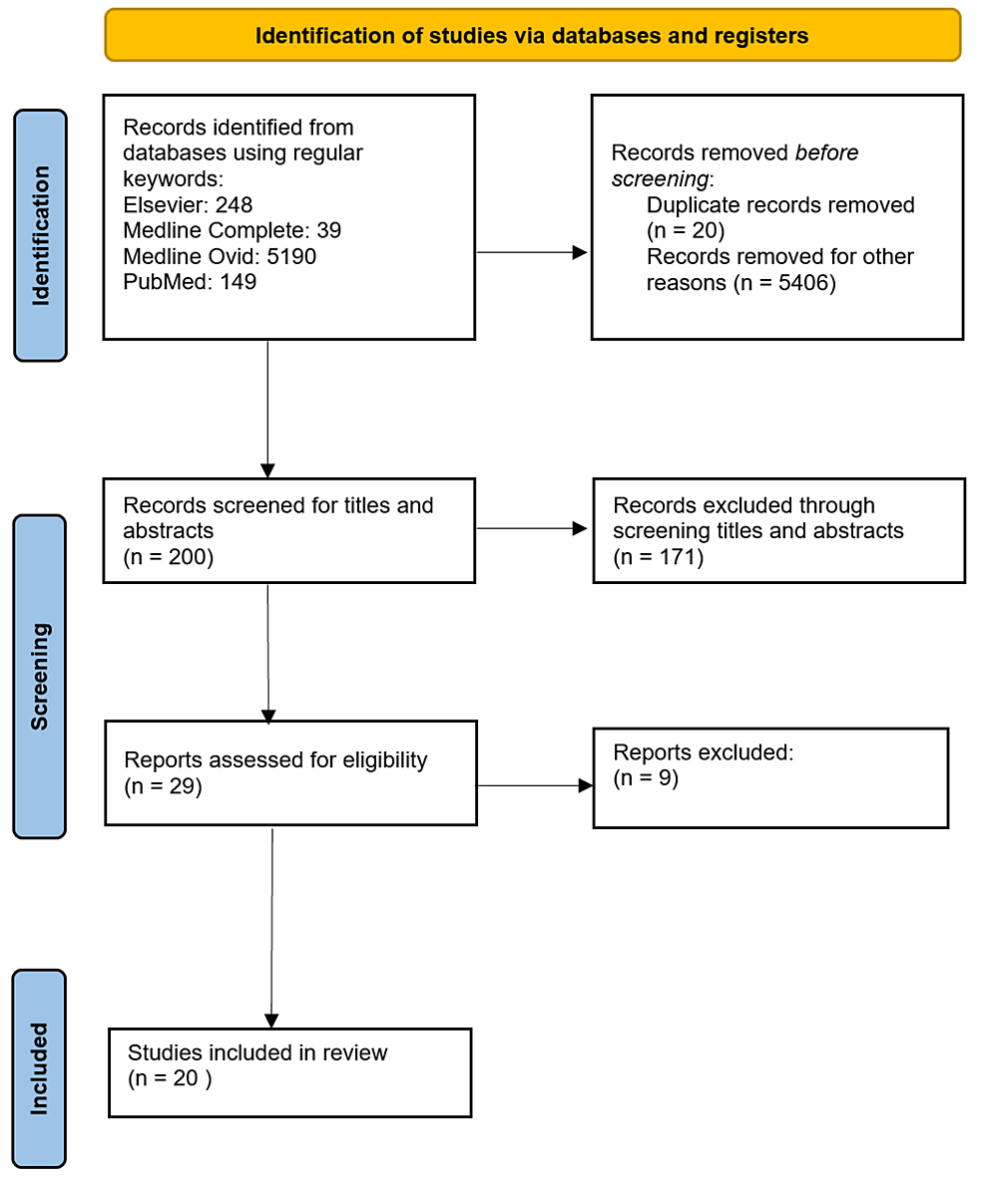
Flowchart of literature review search per PRISMA 2020 guidelines. PRISMA, Preferred Reporting Items for Systematic Reviews and Meta-analysis

A total of 29 relevant articles were found from Medline Complete, Elsevier, Medline and PubMed using the regular keywords. The titles and abstracts of 29 articles were screened, and only 20 articles were relevant to this research topic and met the criteria using standardized quality assessment tools. The PRISMA flowchart of the literature and the search strategy of the studies is shown in Figure 1 [11].

The following data was extracted from each article: authors, year, study design, drug intervention, number of patients, age (years), gender, and concurrent conditions present in the patient. This is summarized in Table 2.

**Table 2:** Study design and patient demographics DVT, deep venous thrombosis; IVIG, intravenous immunoglobulin; N/A, not available; RA, rheumatoid arthritis; ANA, antinuclear antibodies; Ab, antibodies; PTX, pentoxifylline; PCV, polycythemia vera; SLE, systemic lupus erythematosus; VI, venous insufficiency; PAD, peripheral artery disease; DM, diabetes mellitus; HTN, hypertension, COPD, Chronic obstructive pulmonary disease ^a^ 40 patients were in this study; however, 20 patients were given rivaroxaban therapy.

S. No.	Author	Year	Study Design	DRUG intervention	LV Cases	Age (years)	Females	Males	Concurrent conditions present
1.	Leisenring et al. [12]	2020	Case report	Rivaroxaban	1	59	1	0	SLE, breast cancer, and cerebrovascular disease
2.	Franco Marques et al. [13]	2018	Case report	Rivaroxaban	4	37-47	1	0	DVT, heterozygous mutation of Leiden’s factor V and protein S deficiency in two patients
3.	Chen et al. [14]	2017	Case report	Rivaroxaban	3	30-39	1	0	Chickenpox in one patient
4.	Weishaupt et al. [15]	2016	Proof of Concept Trial	Rivaroxaban	20	39–67	18	7	Prothrombotic states/secondary in 11 patients
5.	Drerup et al. [16]	2017	Case report	Rivaroxaban	1	49	0	1	N/A
6.	Evans et al. [17]	2015	Case report	Rivaroxaban	1	57	1	0	None
7.	Lee et al. [18]	2019	Single-centre retrospective observational study	Rivaroxaban	40^a^	N/A	29	11	PCV, VI, coagulation abnormalities in 17 patients
8.	Lee et al. [19]	2016	Case series	Rivaroxaban	3	41-44	1	2	None
9.	Miguel et al. [20]	2019	Case report	Rivaroxaban	1	32	1	0	Ex-smoker
10.	Monshi et al. [21]	2014	Retrospective observational study	IVIG	11	19-60	7	4	DM: two patients; HTN, RA, COPD: two patients; ANA: four patients; Homozygous mutation of Leiden’s factor V: one patient
11.	Ozden et al. [22]	2019	Case series	IVIG	9	30-68	7	0	Stroke in one patient, HTN, DM
12.	Takahagi S.et al. [23]	2021	Case report	IVIG	1	60	0	1	N/A
13.	Bounfour et al. [24]	2013	Case report	IVIG	5	21-73	4	1	None
14.	Pitarch et al. [25]	2004	Case report	IVIG	1	19	1	0	None
15.	Kim et al. [26]	2015	Case report	IVIG	7	17-43	6	1	Two patients with ANA Ab
16.	Kofler et al. [27]	2021	Retrospective observational study	IVIG	25	66 (avg)	15	10	Coagulation disorder in seven patients, Two patients with SLE, HTN, DM, PAD, RA, thrombosis
17.	Yoshioka et al. [28]	2018	Case report	IVIG	1	51	1	0	SLE
18.	Yachoui et al. [29]	2019	Case report	IVIG	1	49	1	0	Prothrombin G20210A, smoker
19.	Dinescu et al. [30]	2021	Case report	IVIG	1	41	1	0	None
20.	Winchester et al. [10]	2015	Case report	Rivaroxaban	2	52-54	2	0	Elevated lipoprotein A level in one patient

Quality Assessment 

Twenty verified publications were subjected to a comprehensive quality evaluation using two established instruments: the Joanna Briggs Institute (JBI) Checklist (n = 16) and the Newcastle-Ottawa Checklist (n = 4).

All 14 case reports, two case series, one clinical trial, and three observational studies satisfied the inclusion and exclusion criteria. The requirements for all three observational studies were satisfied by the Newcastle-Ottawa Checklist. On the Newcastle-Ottawa Checklist, the proof-of-concept clinical trial got seven out of a possible eight points. The review included all articles that did well on the JBI Checklist and the Newcastle-Ottawa Checklist.

Discussion

To further understand the effectiveness of rivaroxaban and IVIG therapy in the treatment of LV, 20 previously published articles with a total of 138 patients diagnosed with LV, were looked at.

Mechanism of Action of Rivaroxaban

Rivaroxaban is a direct factor Xa inhibitor used to prevent stroke in atrial fibrillation as well as to treat and prevent venous thromboembolism [12]. It is a medicine with predictable pharmacokinetics and pharmacodynamics, as well as possessing a fast onset of action. Fortunately, the medicine does not need frequent coagulation monitoring or dose modifications, (for example, based on age, gender, or body weight) [13]. Rivaroxaban has often been the therapy of choice in recent years, owing to the benefit of oral administration and the lack of need for international normalized ratio follow-up, which promotes patient compliance [2]. Rivaroxaban improves patients' quality of life and compliance by being an injection-free option to low molecular weight heparin and a monitoring-free alternative to warfarin [14].

Dosing and Combination Therapy

The dose of rivaroxaban used to treat LV is 10-20 mg daily. Treatment can be started at 10 mg oral rivaroxaban twice per day and could be altered to respond to the variable disease activity of LV [15]. In the review of the 10 cases, the duration of treatment was 2-14 months. Rivaroxaban was started at 20 mg once daily for a patient with SLE. Within two months of treatment, the patient's foot discomfort and discoloration were gone, and she no longer needed narcotic pain medication [12]. Combination treatment also proves effective in the treatment of LV. In a study by Drerup et al., the patient was treated with heparin for four weeks followed by rivaroxaban. The patient did not experience any further relapses under prolonged medication with rivaroxaban 10 mg/day [16]. However, it is unknown whether continuation with another drug such as enoxaparin would have had a good treatment effect by itself. In a study by Weishaupt et al., the population which received at least one dose of enoxaparin did not appear to have better treatment outcomes than the population treated with rivaroxaban alone [15]. This raises the question of whether combination therapy is more effective than monotherapy. Per this review, out of the 56 patients who took rivaroxaban, 48 (85.7%) received monotherapy.

Efficacy of Rivaroxaban

Ten studies focused on the efficacy of rivaroxaban in the treatment of LV. In one of the first-ever registered multicentre proof-of-concept clinical trials, results showed that antithrombotic therapy with rivaroxaban for LV was effective for the amelioration of clinical symptoms such as ulceration and erythema and for improving quality of life. In fact, results showed that pain was reduced by 50% within the first 11 days after treatment [15]. In a study by Franco et al., rivaroxaban effectively decreased pain and cutaneous ulcerations in only a few weeks and was well tolerated by patients [13]. Furthermore, a patient's discomfort had significantly decreased and several of her ulcerations disappeared after a two-month follow-up. On physical examination, there were no signs of infection and merely residual scars. The improvement shown on rivaroxaban was much superior to any prior therapy the patient had had, and it remained effective after eight months of treatment, with all previous ulcers completely healed [17]. In a single-center retrospective study, 15 patients achieved 50% improvement in the median 7.8 weeks after treatment [18]. Rivaroxaban certainly proves efficacious in the treatment of LV. In a study by Lee et al., three patients had a mean of 11 ulcerated lesions prior to rivaroxaban administration. These disappeared after receiving treatment with rivaroxaban for two and a half months [19].

Relapse of Symptoms

Out of the 56 patients who received rivaroxaban, 21.4% relapsed. In a study by Miguel et al., there was no recurrence of symptoms during a follow-up of four months [20]. It was observed that a patient who received treatment with rivaroxaban did not experience any new ulcerations or episodes of pain for 14 months while being treated [16]. However, during the analysis, it was observed that patients did experience some form of relapse after treatment was stopped. In a study of 20 patients taking rivaroxaban, 10 out of 13 patients experienced relapse after the discontinuation of treatment; however, severity was lower than the previous episodes and all relapses were well controlled after re-treatment [18]. In a study by Lee et al., after three months of treatment, the patient had a relapse of ulcer and pain, owing to occupational overwork, but afterward, the patient was free of symptoms and lesions for 11 months [19]. In a separate study, a patient's discomfort subsided after two days, and the ulcers healed within one week. Drowsiness made her therapy harder and, therefore, rivaroxaban was halted. After a week, her discomfort and ulcers came back. At her follow-up consultation, rivaroxaban was resumed at 10 mg per day, resulting in an improvement in her discomfort and the healing of her ulcers [10].

Mechanism of Action of IVIG

Despite the fact that the mechanism of action of IVIG is not completely known, IVIG causes cytokine modulation, pathogen neutralization, complement-mediated damage inhibition, and Fas receptor blocking [8]. According to one theory, IVIG may have anticoagulant effects by inhibiting the thrombogenic effects of antiphospholipid antibodies, inhibiting platelet adhesion, and modulating endothelial function [21]. In recent years, various pathways for the efficacy of IVIG have been discussed, but it can be agreed upon that IVIG does interact with constituent parts of the immune system [22]. Furthermore, IVIG may exert unique immunological and/or anti-thrombotic actions different from other agents. According to a review of 10 studies, the use of IVIG for acute flare-ups may suppress disease activity and cause a longer remission in instances where LV is resistant to anticoagulants and corticosteroids [23].

Dosing and Combination Therapy 

The dose of IVIG administered to patients with LV ranges from 1-2 g/kg, in four-week cycles. In a study by Takahagi et al., patients saw a dramatic decrease in severe pain and ulcerations within four weeks after the first administration of 1.75 g/kg IVIG [23]. Similarly, IVIG was given at a daily dose of 1 g/kg on two consecutive days every four weeks [24]. It's worth noting that effective therapy of LV has also been documented, when IVIG was given at a starting dosage of 2 g/kg, followed by maintenance doses of 1-2 g/kg every four to eight weeks [25]. A treatment protocol of IVIG administered at 2 g/kg, divided over three to five days, repeated for two to three cycles monthly, has also been successful in alleviating pain and ulceration [26]. With regards to combining treatments, in one study by Kofler et al., the majority of patients received antiplatelet aggregation inhibitors or oral anticoagulants in addition to IVIG therapy, primarily for cardiovascular diseases such as coronary artery disease or atrial fibrillation. Therefore, it is not possible to rule out a synergistic impact between anticoagulants and IVIG therapy [27]. Additionally, a study by Yoshioka et al. found that the addition of warfarin to the treatment plan helped the patient's leg ulcerations improve [28]. It was also noted that refractory LV can be managed well with annual IVIG with/without concomitant conventional treatment [26]. This sheds light that IVIG can be used as a monotherapy, and it can still be effective.

Efficacy of IVIG

Furthermore, 10 different studies have demonstrated the efficacy of IVIG in the treatment of LV. In a study by Bounfour et al., IVIG resulted in the healing of ulceration and offered the patient pain relief [24]. In a difficult-to-treat refractory case, the patient had a full remission just after three treatments. These treatments lasted for a period of one year [29]. With the use of IVIG, a positive therapeutic response in terms of ulceration, pain, and daily life constraints was seen, along with acceptable tolerability [27]. In fact, it was shown that four weeks after the first infusion, the ulcers had totally healed, and the pain had vanished for the first time since the onset of the condition [25]. Additionally, in a case of long-term remission of severe LV, the patient achieved remission for seven years, until a loss to follow-up [23]. Serious adverse effects are uncommon and often minimal during IVIG therapy. Headache, hypertension, flushing, fever, nausea, vomiting, and dizziness have been reported among patients [27]. The success of IVIG treatment should not be overlooked and this treatment option should be made more widely available to patients.

Relapse of Symptoms

Out of the 62 patients who received IVIG, 20 patients experienced some form of recurrence of symptoms. Relapse occurred in three patients in a study by Bounfour et al. (median time to relapse: 10.7 months), two of whom were successfully treated using the same technique of therapy. The third patient was treated with dapsone [24]. Additionally, at the end of a study's six-month follow-up period, two patients experienced an immediate relapse. However, the severity was lower, and managing the disease was easier than the first treatment period [22]. This suggests that although relapse occurred, IVIG did help in improving overall disease presentation. In a study by Pitarch et al., no relapse occurred in a patient four weeks after the initial infusion [25]. It was also noted that a patient taking IVIG experienced a long-term remission of up to seven years [23].

Comorbid and Concurrent Conditions

Patients with hypercoagulation syndromes such as factor V Leiden gene mutation, prothrombin G20210A, and protein C and S deficiency usually develop livedoid vasculopathy. There is an established link between livedoid vasculopathy and hypercoagulable disorders [10]. In reality, hypercoagulability due to thrombophilia is a potential cause of LV, and screening for coagulation factor deficits is required [30]. In this analysis, prothrombic states and coagulation abnormalities were observed in many patients as shown in Table 2. Interestingly, patients with LV are more likely to have disorders such as mixed connective tissue, polyarteritis nodosa, SLE, systemic scleroderma, rheumatoid arthritis, and hyperhomocysteinemia, which are all linked to endothelial damage [30]. One of the patients studied in this review presented with SLE and LV.

Rivaroxaban and IVIG Therapy and Treatment Response

Although rivaroxaban and IVIG have two completely different mechanisms of action, they both have been shown to be good therapeutic options in this systematic review. For example, after courses of IVIG healed most of the patient’s ulcers, little improvement was made in her pain. Rivaroxaban was initiated and both the pain and the size of the ulcers improved quickly as a result of this treatment [10]. In another study, several treatments, including aspirin, systemic corticosteroid, pentoxifylline, and immunoglobulins were used, but the symptoms and skin lesions waxed and waned without complete remission. Rivaroxaban treatment resulted in clinical improvement in all three patients [19]. In a study by Chen et al., rivaroxaban was found to be more tolerable than earlier medications [14]. A study by Ralph et al., on the other hand, found that a variety of therapies, including rivaroxaban, antiplatelet drugs, oral steroids, warfarin, pentoxifylline, and, more recently, rituximab (for one year), were tried with no success. After three infusions of IVIG at a dosage of 1 g/kg monthly, the lesions were completely resolved [29]. In a study by Ozden et al., before initiating IVIG therapy, the patients had been treated with the following treatment options without any remission or healing of ulcers: prednisone, azathioprine, mycophenolate mofetil, mycophenolate sodium, and aspirin-clexane-warfarin sodium-pentoxifylline. All patients revealed significant healing with IVIG therapy [22]. 

**Figure 2:**
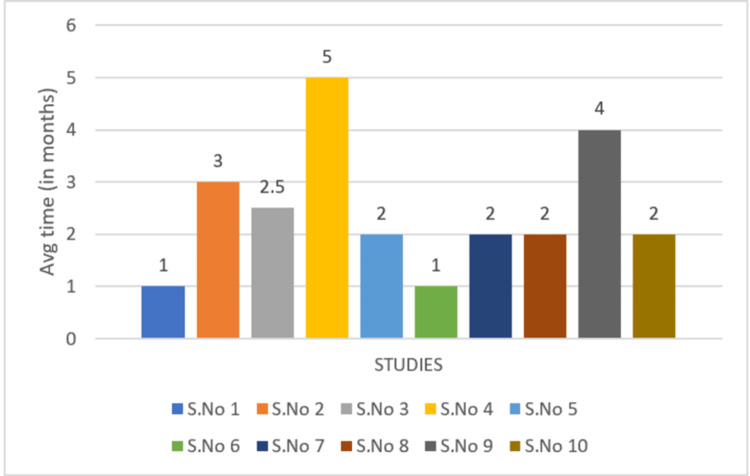
Treatment response time of rivaroxaban S.No 1: Franco Marques et al. [13]; S.No 2: Weishaupt et al. [15]; S.No 3: Lee et al. [19]; S.No 4: Miguel et al. [20]; S.No 5: Drerup et al. [16]; S.No 6: Winchester et al. [10]; S.No 7: Leisenring et al. [12]; S.No 8: Evans et al. [17]; S.No 9: Chen et al. [14]; S.No 10: Lee et al. [18] S.No: study number

**Figure 3:**
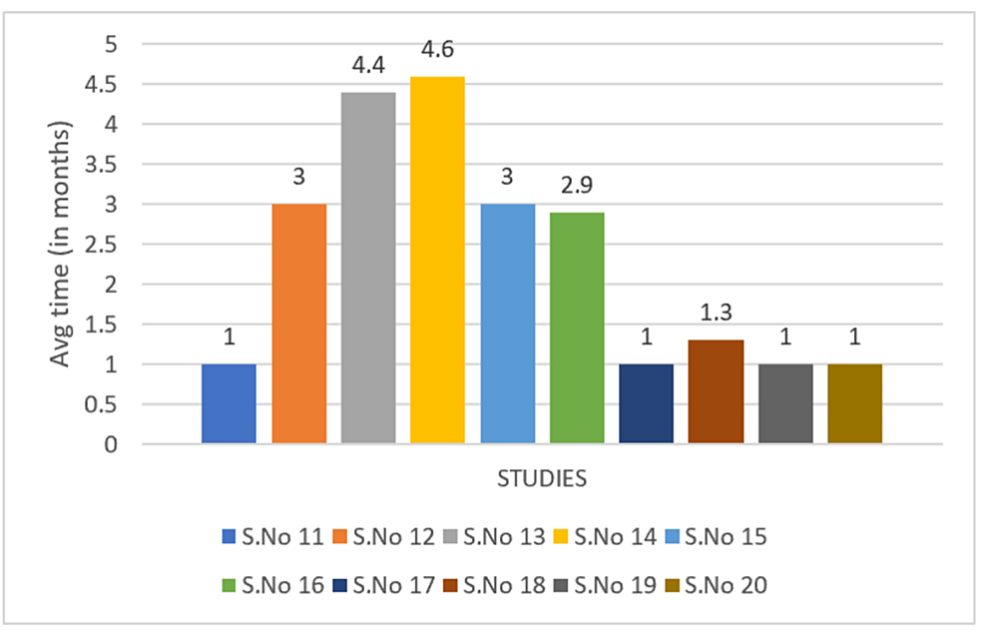
Treatment response time of intravenous immunoglobulins (IVIG) S.No 11: Bounfour et al. [24]; S.No 12: Yachoui et al. [29]; S.No 13: Kofler et al. [27]; S.No 14: Ozden et al. [22]; S.No 15: Dinescu et al. [30]; S.No 16: Monshi et al. [21]; S.No 17: Takahagi et al. [23]; S.No 18: Yoshioka et al. [28]; S.No 19: Kim et al. [26]; S.No 20: Pitarch et al. [25] S.No: study number

Response times were compared, as seen in Figures 2, 3. "Response times" include the first signs of ulcer healing and/or a reduction in pain. Average times were measured in months.

Of note, the average treatment response time was 2.4 months for rivaroxaban versus 2.3 months for IVIG as seen in Figures 2, 3. This suggests that although these drugs came from different classes, treatment response times were almost the same, begging the question of whether LV can be targeted from different mechanisms. 

Limitations 

The limitations of this systematic review include the types of studies presented. The majority of the studies were case studies, case series, and observational studies. The need for large-scale clinical trials is imperative and would reduce the risk of bias when reporting patient outcomes. In this review, the patient population was small, which may not be representative of the general population. Treatment was not long-term, which did not represent a long-term regimen for a long-term chronic disease.

## Conclusions

Both rivaroxaban and IVIG have proven to be efficacious in the treatment of LV. Although LV is a rare condition, this review has revealed the need for more large-scale placebo-controlled clinical trials and better-established therapeutic guidelines. In comparing the efficacy of rivaroxaban and immunoglobulin, a huge potential for monotherapy or even combination therapies is evident. Rivaroxaban has been shown to be an effective drug with good patient compliance. IVIG is a well-tolerated and safe treatment for LV. Future research should focus on the strong therapeutic response shown in the review.
